# RETRACTED: Ferlazzo et al. A Flavonoid-Rich Extract from Bergamot Juice, Alone or in Association with Curcumin and Resveratrol, Shows Protective Effects in a Murine Model of Cadmium-Induced Testicular Injury. *Pharmaceuticals* 2021, *14,* 386

**DOI:** 10.3390/ph19070984

**Published:** 2026-06-25

**Authors:** Nadia Ferlazzo, Antonio Micali, Herbert Ryan Marini, Josè Freni, Giuseppe Santoro, Domenico Puzzolo, Francesco Squadrito, Giovanni Pallio, Michele Navarra, Santa Cirmi, Letteria Minutoli

**Affiliations:** 1 Department of Biomedical, Dental Sciences and Morphofunctional Imaging, University of Messina, 98125 Messina, Italy; nadia.ferlazzo@unime.it (N.F.); amicali@unime.it (A.M.); freni.jose.89@gmail.com (J.F.); giuseppe.santoro@unime.it (G.S.); puzzolo@unime.it (D.P.); 2 Department of Clinical and Experimental Medicine, University of Messina, 98125 Messina, Italy; hrmarini@unime.it (H.R.M.); fsquadrito@unime.it (F.S.); gpallio@unime.it (G.P.);; 3 Department of Chemical, Biological, Pharmaceutical and Environmental Sciences, University of Messina, 98166 Messina, Italy; mnavarra@unime.it; 4 Department of Pharmacy-Drug Sciences, University of Bari “Aldo Moro”, 70125 Bari, Italy

The journal retracts the article titled, “A Flavonoid-Rich Extract from Bergamot Juice, Alone or in Association with Curcumin and Resveratrol, Shows Protective Effects in a Murine Model of Cadmium-Induced Testicular Injury” [1], cited above.

Following publication, concerns were brought to the attention of the Editorial Office regarding the integrity of an image in this article [1].

In accordance with the journal’s standard procedures, the Editorial Office and Editorial Board conducted an investigation that identified indications of inappropriate image editing and duplication between Figure 3A published in [1] and Figure 1C published in [2], presenting different experimental conditions. Although the authors cooperated with the investigation, the explanations and supporting materials provided were not deemed sufficient by the Editorial Board to resolve the identified concerns. Consequently, the Editorial Board has lost confidence in the reliability of the reported findings and has decided to retract this publication.

This retraction was approved by the Editor-in-Chief of the journal *Pharmaceuticals*.

The authors have been informed of this retraction but did not provide a comment on this decision.
